# Synergistic Regulation of Bile Acid-Driven Nitrogen Metabolism by Swollenin in Ruminants: A Microbiota-Targeted Strategy to Improve Nitrogen Use Efficiency

**DOI:** 10.3390/ani16010149

**Published:** 2026-01-05

**Authors:** Lizhi Li, Haibo Zhang, Linfei Zhan, Weikun Guan, Junhao Hu, Zi Wei, Wenbo Wu, Yunjing Wu, Qingfeng Xing, Jianzhong Wu, Zhen Li, Qin Liu, Jifa Chen, An Yuan, Dongsheng Guo, Kehui Ouyang, Jiarui Yang, Wei Hu, Xianghui Zhao

**Affiliations:** 1 School of Life Sciences and Environmental Resources, Yichun University, Yichun 336000, China; 190033@jxycu.edu.cn (L.L.); zhanghaiboainide@163.com (H.Z.); zlf321006@163.com (L.Z.); 999342@jxycu.edu.cn (W.G.); 13197943508@163.com (J.H.); 18736803981@163.com (Z.W.); a_1090639975@163.com (W.W.); 18296398581@163.com (Y.W.); 15178916656@163.com (Q.X.); tianlangxing9091@126.com (J.W.); zhenli1995@sina.com (Z.L.); liuqin675@hotmail.com (Q.L.); jifachen66@jxycu.edu.cn (J.C.); fzlizhi001@163.com (A.Y.); gds960016@126.com (D.G.); 2 Jiangxi Province Key Laboratory of Animal Nutrition, Animal Nutrition and Feed Safety Innovation Team, College of Animal Science and Technology, Jiangxi Agricultural University, Nanchang 330045, China; oykh@jxau.edu.cn (K.O.); yangjiaruiaa@163.com (J.Y.); 3 Research Center for Traditional Chinese Veterinary Medicine and Animal Embryo Engineering Technology, Yichun University, Yichun 336000, China

**Keywords:** Swollenin, young goats, gut microbiota, bile acid metabolism, nitrogen metabolism

## Abstract

The annual nitrogen loss from the livestock production sector poses a significant threat to the global natural environment. Therefore, it is urgent to focus on improving the nutrient utilization efficiency of ruminants and promoting the sustainable development of livestock production. Twelve 60-day-old Ganxi goats with similar body weights were selected and randomly assigned to two dietary treatment groups. The control group was fed only a basal diet, while the treatment group was supplemented with 32 mg/d of Swollenin. The experiment lasted for 30 days. At the end of the experimental period, the goats were euthanized, and their intestinal contents were collected, rapidly frozen, and stored at −80 °C for subsequent metagenomic and metabolomic analyses. In the Swollenin group, we observed changes in gut microbiota structure and significantly enhanced feed conversion efficiency compared to the control group. Notably, genera such as *Bacteroides*, *Ruminococcus*, and *Bifidobacterium* exhibited significantly higher abundance. Following Swollenin supplementation, the gene abundance associated with the secondary bile acid biosynthesis pathway in the intestinal tract of young goats was significantly higher. The levels of primary bile acids (BAs), including taurocholic acid, glycocholic acid, taurochenodeoxycholic acid, and glycochenodeoxycholic acid, were significantly lower, while the concentrations of secondary BAs such as ursodeoxycholic acid and deoxycholic acid were significantly higher. The abundance of nitrogen-fixing and nitrogen-assimilating genes in the gut of young goats in the Swollenin group was significantly higher. Furthermore, co-occurrence network analysis revealed a strong correlation between bile acid metabolism and nitrogen metabolism pathways. These results suggest that nutritional regulation may serve as a preventive strategy to optimize the symbiotic development of animals and their gut microbiota, ultimately improving nitrogen utilization.

## Simple Summary

Ruminant production plays a pivotal role in the global nitrogen cycle. Improving the nitrogen conversion efficiency in the hindgut of ruminants is of great significance for enhancing nutrient utilization efficiency and maintaining production performance. Recent research has demonstrated that bile acid metabolism in the ruminant intestine may indirectly regulate nitrogen utilization by modulating the composition and functional characteristics of microbial communities involved in nitrogen assimilation and ammonification processes. Meanwhile, the establishment of gut microbiota in young ruminants profoundly affects growth performance and long-term health. Consequently, targeted interventions on the gut microbiota–bile acid axis could offer a novel strategy to improve nitrogen use efficiency in ruminants. Previous studies have indicated that Swollenin reshapes the microbial community structure and alters the abundance and metabolic activity patterns of key metabolic enzymes during in vitro rumen fermentation. The present study further revealed that Swollenin can optimize the gut microbial composition of young goats. Specifically, it significantly improved feed conversion efficiency, promoted primary bile acids to secondary bile acids biotransformation, and notably increased the abundances of nitrogen fixation and assimilation genes. Furthermore, co-occurrence network analysis confirmed a strong correlation between the bile acid metabolic pathway and the nitrogen metabolic pathway.

## 1. Introduction

The global nitrogen cycle is fundamental to planetary biochemistry, influencing agricultural productivity, ecosystem stability, and climate dynamics [1,2]. Efficient nitrogen utilization is critical for sustaining food production to support a growing human population and for reducing the environmental consequences of reactive nitrogen loss. Excess nitrogen emissions primarily occur as ammonia volatilization and nitrous oxide, with nitrous oxide being a potent greenhouse gas that contributes significantly to global climate change, possessing a global warming potential 150 times that of carbon dioxide over a 100-year period [3,4]. Annual nitrogen emissions from livestock production are estimated at approximately 65 teragrams, representing one-third of total human-induced nitrogen emissions [5]. Consequently, enhancing our understanding of nitrogen transformation and retention in major ecosystems—especially within intensive livestock production systems—is crucial for developing effective greenhouse gas emission reduction strategies.

Ruminant livestock production plays a vital and intricate role in the global nitrogen cycle. Projections indicate that by 2050, changing patterns in ruminant production will lead to decreased efficiency in both production and nutrient utilization [6]. This inefficiency is primarily evident in the form of urea excreted in ruminant urine, which quickly hydrolyzes into ammonia, significantly contributing to atmospheric NH_3_ emissions [7]. These emissions are estimated to account for roughly 40% of global anthropogenic emissions, creating a considerable environmental burden [8]. Unless there is concerted global action to reduce ruminant meat consumption to mitigate greenhouse gas emissions, enhancing feed efficiency remains the most viable strategy. This improvement could potentially reduce nitrogen excretion from ruminants by 10% [6]. Therefore, increasing the rate of nitrogen conversion in the ruminant digestive tract is essential for minimizing the environmental impact of livestock production while maintaining productivity.

The intestinal phase of the ruminant digestive system, especially the hindgut, is critical for nitrogen cycling, absorption, and fixation processes [9]. However, a significant portion of dietary nitrogen consumed by ruminants is excreted rather than utilized for tissue development [10]. Recent studies suggest that bile acids (BAs), particularly secondary BAs produced by gut microbiota, not only serve as lipid emulsifiers but also act as influential signaling molecules that regulate host metabolism and gut microbial ecology [11]. Importantly, secondary BAs have microbially modulatory properties that affect various enzymatic activities in the gut related to nitrogen metabolism, including bile salt hydrolase (BSH) and urease [11]. Emerging evidence indicates that microbiota-mediated modulation via BAs might indirectly influence nitrogen utilization by affecting the composition and function of microbial communities involved in nitrogen assimilation or ammonification [12]. This suggests a potentially significant, though underexplored, link between host–microbial co-metabolism and nitrogen processing in the gut. Consequently, these findings suggest that interventions targeting the gut microbiota–BA axis could indirectly enhance nitrogen metabolism and retention in ruminants, offering a novel strategy to improve nitrogen use efficiency.

Swollenin, a protein derived from *Trichoderma reesei*, shares structural similarities with plant expansins and is known for its ability to swell and partially disrupt cellulose structures [13]. Previous research has primarily examined Swollenin’s effects on the disintegration of fibrous materials in fermentation environments, which accelerates microbial fiber degradation and energy production [14]. However, alterations in fermentation types can reshape the microbial environment, and shifts in host gut microbiota can also modify the abundance and migration patterns of various metabolism-related enzymes [15,16]. In our earlier study, we introduced heterologously expressed Swollenin protein into an in vitro fermentation experiment simulating the ruminant rumen. The results showed a significant increase in the abundance of *Bacteroides*, *Clostridium*, *Lactobacillus*, *Bifidobacterium*, and *Ruminococcus*, which are crucial for secondary BAs production, thereby altering the microecological structure [14]. Additionally, young ruminants are influenced by feeding systems, nutritional regulation, and various early growth factors. The establishment of their gut microbiota has a profound impact on both growth performance and long-term health. For example, modifying the degradation of dietary nutrients can directly alter the structure and metabolic profiles of the intestinal and fecal microbiota in sheep, thereby enhancing nitrogen absorption [17]. This underscores the intricate network involved in regulating nitrogen metabolism in ruminants, encompassing dietary interventions and interactions between microbes and hosts.

In light of these findings and the emerging relationships among host gut microbiota, BAs, and nitrogen metabolism, this study aims to explore the application of Swollenin in ruminant production. We utilized metagenomic sequencing and targeted metabolomics to analyze changes in gut microbiota structure and bile acid metabolism profiles in young goats. Additionally, we examined the evolution of gene abundance in nitrogen metabolism pathways within the gut environment. Following this, we will perform a coexistence analysis to determine whether Swollenin can indirectly influence the abundance of nitrogen metabolism-related genes in ruminants by bridging the gut microbiota–bile acid co-metabolism axis. This research enhances our understanding of using innovative products to improve livestock production and nitrogen utilization efficiency in ruminants, providing valuable insights for fostering the sustainable development of global animal husbandry.

## 2. Materials and Methods

### 2.1. Swollenin Production, Animal Trial Design, and Housing

The synthesis method and characteristics of the Swollenin protein utilized in this study are detailed in previous publications from our laboratory [14]. In brief, the coding sequence of *Swollenin* used in this experiment was derived from *Trichoderma reesei* RutC-30 (GenBank accession AJ245918.1). The *Swollenin* gene was optimized according to the codon preference of *Pichia pastoris*, and the optimized sequence can be found in our laboratory’s previous study [14]. Gene synthesis was commissioned to GenScript Nanjing Co., Ltd. (Nanjing, China). The synthesized DNA was ligated into the pPICZαA vector to construct the recombinant plasmid pPICZαA-*Swollenin*, which was verified by PCR amplification, restriction enzyme analysis, and sequencing. The gene-specific primers used were swoF (CAACAAAACTGTGCTGC) and swoR (ATTTTGAGAGAATTGAACAC). After linearization, the recombinant plasmid pPICZαA-*Swollenin* was transformed into *Pichia pastoris* X33 competent cells. Primary screening of target strains was performed using YPD agar plates containing zeocin, and positive colonies were confirmed by PCR. The verified transformants were induced for expression with methanol in BMMY medium. After concentrating the supernatant, the expression of Swollenin was detected by SDS-PAGE. According to Invitrogen’s *Pichia pastoris* Fermentation Process Guidelines, the production of Swollenin was carried out in a vertical glass bioreactor (Minifors 2, Infors HT, Bottmingen, Switzerland) with a working volume of 4.0 L. After fermentation, the supernatant was collected by centrifugation and further concentrated. The concentrated supernatant was subjected to standard chromatography using a Bio-Scale Mini Nuvia IMAC Ni-Charged column (Bio-Rad Laboratories, Inc., Hercules, CA, USA) to obtain purified Swollenin protein. The concentrated protein sample was verified by Western blot, and the results are available in previous study [14].

Animal experiments were conducted in accordance with the principles and operational guidelines established by the Yichun University Institutional Animal Care and Use Committee (ID number: JXSTUDKY2023014). The study adhered to guidelines set forth in the revised Animals (Scientific Procedures) Act 1986 (UK) and the European Directive 2010/63/EU.

A total of twelve Ganxi goats (males, 60 ± 3 days old; 6.15 ± 0.48 kg body weight) were randomly assigned to two dietary treatments, with six goats per group. The basic diet was prepared according to the NRC (2021) nutrient requirements for growing goats (Table S1). Following a 7-day adaptation period to the basic diet, the control (CON) group received only this diet, while the treatment group was supplemented with 32 mg/d of Swollenin. The supplementation dosage of Swollenin was referenced from our previous study [14]. The goats were fed daily at 7:00 a.m. and 6:00 p.m. in individual cages with ad libitum access to water and feed. The trial continued for 30 days.

### 2.2. Growth Performance and Sample Collection

Goat body weight was recorded on day 0 and day 30 prior to the morning feeding. Average daily gain (ADG) was calculated using the formula: (final weight − initial weight)/30. Daily dry matter intake (DMI) was calculated according to relevant previous literature as the total feed offered minus the amount refused [18]. The feed conversion ratio (F/G) was determined by dividing DMI (kg/day) by ADG (kg/day). Four hours post-feeding on day 30, all goats were euthanized via exsanguination under sodium pentobarbital anesthesia (30 mg/kg body weight). Intestinal feces were collected, rapidly frozen in liquid nitrogen, and stored at −80 °C for subsequent metagenomics and metabolomics analysis.

### 2.3. DNA Extraction and Metagenomic Sequencing

Microbial genomic DNA was extracted from goat intestinal fecal samples using the E.Z.N.A. Stool DNA Kit (Omega Biotek, Hercules, CA, USA). DNA concentration and purity were assessed using TBS-380 (Turner Biosystems, Inc., Sunnyvale, CA, USA) and NanoDrop2000 (Thermo Fisher Scientific, Wilmington, DE, USA), while DNA integrity was evaluated through 1% agarose gel electrophoresis.

Metagenomic sequencing was performed using the Illumina NovaSeq platform at Shanghai Majorbio Biopharm Technology Co., Ltd. (Shanghai, China). The DNA was fragmented to a size of 350 bp using the Covaris M220 (Covaris, Inc., Woburn, MA, USA). Filtered sequences were matched to the host genome (https://www.ncbi.nlm.nih.gov/genome/?term=txid9925[orgn], accessed on 1 December 2023), resulting in clean reads for the purposes of splicing and assembly. Prodigal (https://github.com/hyattpd/Prodigal, accessed on 3 December 2023) and CD-HIT software (http://www.bioinformatics.org/cd-hit/, accessed on 7 December 2023) were utilized to forecast open reading frames from each assembled contig and to build the non-redundant gene catalog. High-quality sequencing reads were aligned against this catalog (clustered at 95% identity) using SOAPaligner (https://github.com/ShujiaHuang/SOAPaligner, accessed on 10 December 2023), allowing for the calculation of per-gene abundance. The non-redundant gene catalog was then subjected to a homology search against the non-redundant protein sequence database (NR) using BLASTP (http://www.ncbi.nlm.nih.gov/, accessed on 14 December 2023).

Species-level gene abundances were aggregated based on taxonomic annotation from the NR classification database to estimate overall species abundance. Functional annotation of the non-redundant gene catalog was performed against the Kyoto Encyclopedia of Genes and Genomes (KEGG) database (http://www.genome.jp/kegg/genes.html, accessed on 20 December 2023) using sequence similarity searches. Finally, the abundances of KEGG Orthology (KO) and KEGG pathways were established based on the annotated gene abundance.

### 2.4. Targeted Metabolomics Analysis in Bile Acid Metabolism

Samples weighing 25 mg were spiked with 20 μL of internal standard working solution 1 (2000 ng/mL) and 380 μL of extraction solvent (methanol:water = 4:1, *v*/*v*). Cryogenic grinding was performed out at −10 °C and 50 Hz for 6 min, followed by low-temperature ultrasonication at 5 °C and 40 kHz for 30 min. After a static incubation at −20 °C for 30 min, the samples were centrifuged at 13,000 rcf (4 °C, 15 min). A 200 μL aliquot of the supernatant was analyzed using LC-ESI-MS/MS (UHPLC-Qtrap, Waters Corporation, Milford, MA, USA). For chromatographic separation, an ExionLC AD system (SCIEX, Redwood City, CA, USA) was used with a Waters BEH C18 column (150 × 2.1 mm ^2^, 1.7 μm, Waters Corporation, Milford, MA, USA) at 50 °C, with a 5 μL injection volume. The mobile phase consisted of A: 0.1% formic acid in water and B: 0.1% formic acid in acetonitrile. Mass spectrometric detection was performed on an AB SCIEX QTRAP 6500+ (SCIEX, Redwood City, CA, USA) in negative mode with the following parameters: curtain gas 35, collision gas medium, ionspray voltage −4500 V, temperature 550 °C, ion source gas1 50 psi, and ion source gas2 50 psi.

Quantitative analysis was performed using AB Sciex OS software (Version 3.x), which employed default parameters for the automatic identification and integration of ion fragments, with additional manual verification. Calibration curves were created by plotting the peak area ratio of the analyte to the internal standard on the y-axis against the analyte concentration on the x-axis. This process enabled the generation of linear regression models. Sample concentrations were then determined by substituting the measured peak area ratio of the sample analyte to the internal standard into these linear equations.

### 2.5. Statistical Analysis and Mapping

Experimental data were analyzed using IBM SPSS Statistics (v20.0) software with the *t*-test. Data are presented as mean ± standard deviation, and the significance level was set at *p* < 0.05. For metagenomic and metabolomic data analyses, the Wilcoxon rank-sum test was used with a significance level of *p* < 0.05 (marked as *) and *p* < 0.01 (marked as **), and false discovery rate (FDR) was applied for multiple test correction. The Linear Discriminant Analysis (LDA) Effect Size (LEfSe) algorithm was applied to identify differentially abundant features, using thresholds of *p* < 0.05 and LDA scores ≥ 2.5. Spearman correlation matrices were visualized through heatmaps created with the R heatmap package (v3.2.5). Beta-diversity patterns were illustrated using Principal Coordinate Analysis (PCoA), with two-dimensional projections generated via R’s mapping tools (v3.2.5). Network analysis was performed on the Gephi platform (Version 0.10.1) to quantify associations within the co-occurrence network involving nitrogen-cycling pathways, secondary bile acid metabolism-related genes, and microbial communities, utilizing Spearman’s rank correlation coefficients. Diagrams depicting bile acid metabolism pathways were sourced from the Figdraw database.

## 3. Results

### 3.1. Effect of Swollenin on Goat Growth Performance

In this study, the dietary inclusion of Swollenin led to subtle but consistent improvements in the growth performance of young goats (Table 1). Although final body weight (8.43 ± 0.25 kg) and average daily gain (72.47 ± 8.14 g d^−1^) were numerically higher than in the control group (+3.8% and +5.7%, respectively), these differences did not reach statistical significance (*p* = 0.104 and 0.290). Importantly, Swollenin significantly improved feed efficiency, evidenced by a 3.8% reduction in daily dry matter intake (263.1 vs. 273.6 g, *p* = 0.049), and an 8.7% improvement in the feed-to-gain ratio (3.67 vs. 4.02, *p* = 0.082).

### 3.2. Effect of Swollenin on Gut Microbiota Changes in Young Goats

**Figure 1:**
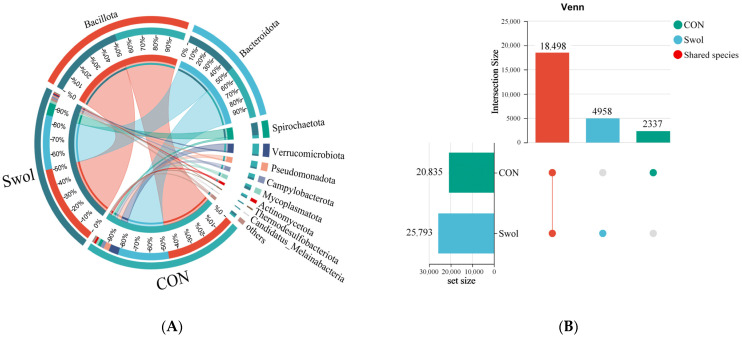

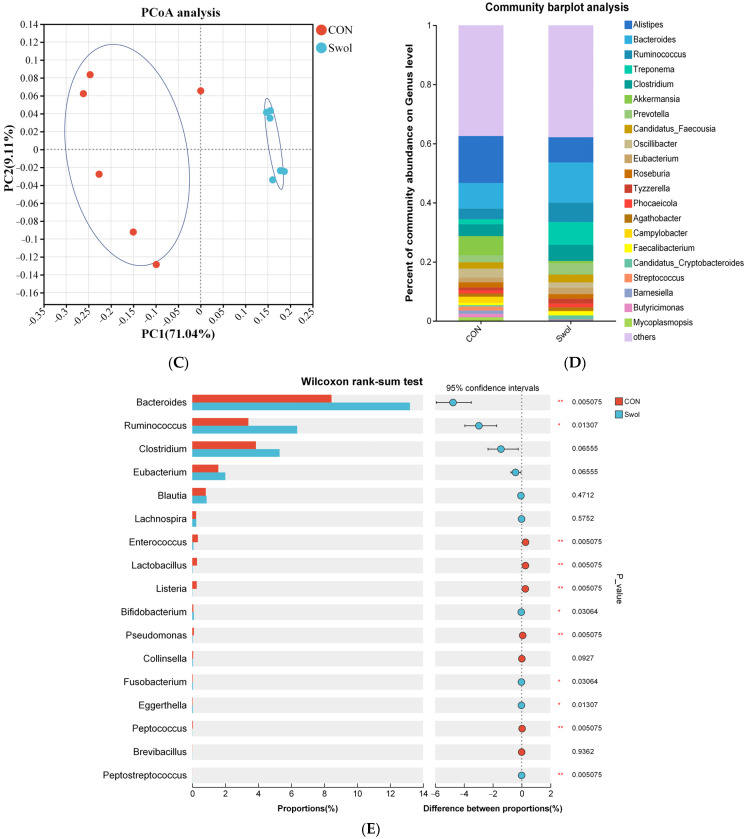
Impact of Swollenin on the top 10 bacterial phyla (**A**), the number of common or unique species (**B**), the PCoA of microbiota at the species level ((**C**), with grouping ellipses indicating sample clusters of each group), the microbiota composition at the genus level (**D**), and the differences in the abundance of bile acid-metabolizing bacteria at the genus level (**E**). Asterisk symbols indicate statistical significance: * (*p* < 0.05), ** (*p* < 0.01). CON, control group; Swol, swollenin group; PCoA, principal coordinate analysis.

Co-occurrence network analysis, illustrated through a Circos plot, revealed phylum-level associations between samples and species abundance in the Swollenin and control groups (Figure 1A). The Bacillota (formerly Firmicutes) and Bacteroidota phyla emerged as the dominant microbiota, with relative abundances of 47.28% and 34.02% in the control group, respectively, increasing to 50.95% and 34.10% in the Swollenin group. After young goats ingested Swollenin, the relative abundance of the phylum Spirochaetota was higher, rising from 1.89% to 7.84%. Conversely, the relative abundances of the Pseudomonas and Verrucomicrobiota phyla in the control group were 3.60% and 6.53%, respectively, which declined to 0.91% and 0.83% following Swollenin ingestion.

To evaluate the common and unique species across different groups, Venn plot analysis was performed to demonstrate the joint and individual effects of Swollenin intake on goat gut microbiota (Figure 1B). This analysis revealed a total of 18,498 common species when comparing the gut microbiota composition of the control and Swollenin groups at the species level. Additionally, Swollenin intake led to the emergence of 4958 endemic species in the goat gut microbiota, in contrast to the 2337 endemic species identified in the control group. This finding suggests that Swollenin intake enhances the richness of gut microbiota in young goats compared to the control group. Furthermore, PCoA analysis at the species level revealed distinct clustering of the gut microbiota between the Swollenin and control groups, explaining 80.15% of the total variation across all axes (Figure 1C). These results indicate significant differences in both the composition and function of the gut microbiota between the two groups of goats.

At the genus level, this study employed a community bar chart to depict the composition of dominant genera, each with an abundance exceeding 1%, across the two groups (Figure 1D). The results reveal that after supplementation with Swollenin, the relative abundances of the genera *Bacteroides*, *Treponema*, *Ruminococcus*, *Clostridium*, *Prevotella*, and *Eubacterium* in the gut microbiota of young goats were 13.59%, 7.74%, 6.51%, 5.44%, 3.82%, and 2.06%, respectively. In contrast, the relative abundances of these genera in the CON group were 8.72%, 1.70%, 3.54%, 4.02%, 2.32%, and 1.63%, respectively. Notably, the relative abundance of the genus *Akkermansia* was 6.49% in the CON group, whereas it was only 0.79% in the Swollenin group.

### 3.3. Effect of Swollenin on Bile Acid-Metabolizing Bacteria in the Gut of Goats

The Wilcoxon rank-sum test was employed to analyse differences in the average abundance of bile acid-metabolizing bacteria at the genus level among 17 species (Figure 1E). Following swollenin supplementation, the abundances of the genera *Bacteroides*, *Ruminococcus*, *Bifidobacterium*, *Eggerthella*, *Peptostreptococcus*, and *Fusobacterium* in the intestines of young goats were significantly higher than those in the CON group. While the abundance of *Clostridium*, *Eubacterium*, *Blautia*, and *Lachnospira* genera also saw some increase in the Swollenin group, these changes were not statistically significant. In the Con group, the abundances of the genera *Enterococcus*, *Lactobacillus*, *Listeria*, *Pseudomonas*, and *Peptococcus* were significantly higher.

### 3.4. The Effect of Swollenin on the Abundance of Secondary Bile Acid Metabolism-Related Genes in the Gut of Goats

**Figure 2:**
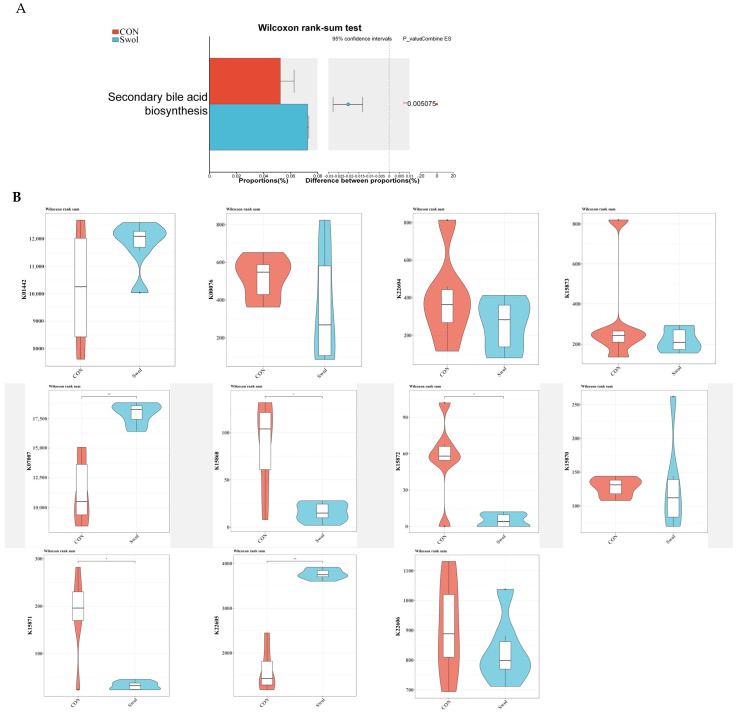
Effect of Swollenin on the bile acid metabolism pathway in the gut of goats. (**A**) The Wilcoxon rank-sum test analyses the activity of the secondary bile acid biosynthesis pathway. (**B**) The differential analysis of core functional orthologs. Asterisk symbols indicate statistical significance: * (*p* < 0.05), ** (*p* < 0.01). CON, control group; Swol, swollenin group.

**Figure 3:**
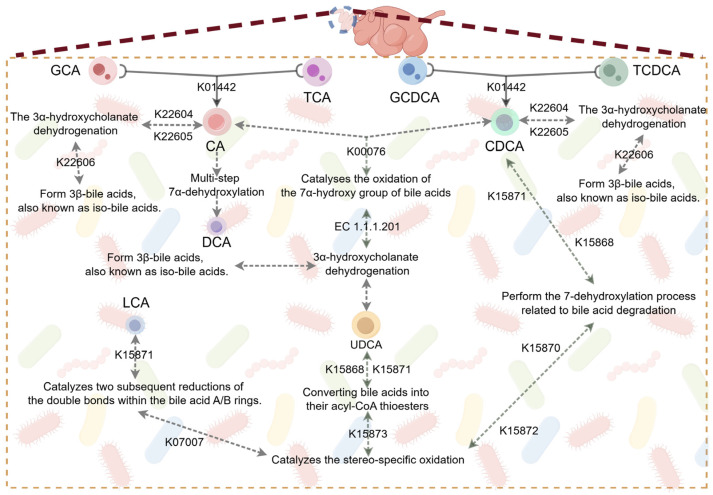
Overview of the Bile Acid Metabolism Process. GCA, glycocholic acid; TCA, tarocholic acid; GCDCA, glycochenodeoxycholic acid; TCDCA, taurochenodeoxycholic acid; CA, cholic acid; CDCA, chenodeoxycholic acid; DCA, deoxycholic acid; LCA, lithocholic acid; UDCA, ursodeoxycholic acid.

The study found that compared with the CON group, the enrichment level of genes involved in the secondary bile acid biosynthesis pathway in the gut of young goats was significantly higher following Swollenin supplementation (Figure 2A). In this study, we conducted a Wilcoxon differential analysis on 11 KOs indicators, which represent key functional orthologs in the secondary bile acid biosynthesis pathway (Figure 2B and Figure 3). We found significant differences among five KOs taxa between the two groups. Among them, the abundances of *K07007* (3-dehydro-bile acid δ4,6-reductase, baiN) and *K22605* (3-α-hydroxycholanate dehydrogenase, baiA) were significantly higher in the Swollenin group. Although the abundance of *K01442* (choloylglycine hydrolase, cbh) in the Swollenin group was higher than that in the CON group, the difference was not statistically significant. In contrast, the abundances of *K15868* (bile acid-coenzyme A ligase, baiB), *K15871* (bile acid CoA-transferase, baiF), and *K15872* (bile-acid 7α-dehydratase, baiE) were significantly higher in the CON group.

### 3.5. The Effect of Swollenin on Intestinal Bile Acid Metabolism in Young Goats

**Figure 4:**
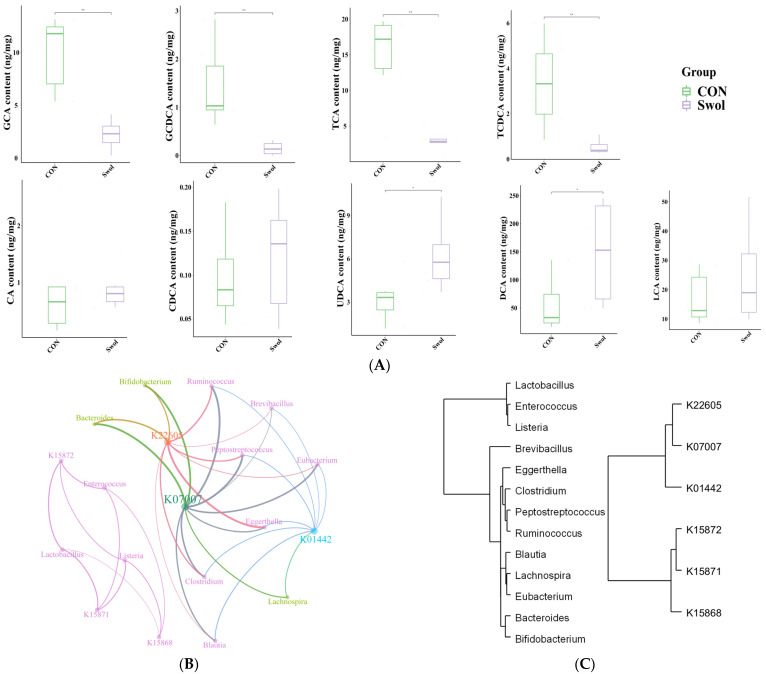
Differential analysis of bile acid concentration (**A**). (**B**) Co-occurring network analysis between core functional orthologs of the secondary bile acid biosynthesis pathway and bile acid-metabolizing bacteria. (**C**) The cluster analysis of bile acid-metabolizing bacteria and core functional orthologs (r > 0.5 and *p* < 0.05); Nodes, node size, and edges repre-sent bacterial communities and core functional orthologs, the degree of nodes, and significant positive correlations, re-spectively. The community detection algorithm (resolution of 1.0) resolves the overall data and assigns different colors to nodes and edges. Asterisk symbols indicate statistical significance: * (*p* < 0.05), ** (*p* < 0.01). CON, control group; Swol, swollenin group; GCA, glycocholic acid; TCA, tarocholic acid; GCDCA, glycochenodeoxycholic acid; TCDCA, tau-rochenodeoxycholic acid; CA, cholic acid; CDCA, chenodeoxycholic acid; DCA, deoxycholic acid; LCA, lithocholic acid; UDCA, ursodeoxycholic acid.

Secondary BAs are produced by the dehydroxylation of primary BAs, a process facilitated by enzymes secreted by gut microbiota that removes hydroxyl groups (Figure 3). In the Swollenin group, the levels of primary BAs, including taurocholic acid (TCA), glycocholic acid (GCA), taurochenodeoxycholic acid (TCDCA), and glycochenodeoxycholic acid (GCDCA), were significantly lower (*p* < 0.05; Figure 4A), while the concentrations of secondary BAs such as ursodeoxycholic acid (UDCA) and deoxycholic acid (DCA) were significantly higher (*p* < 0.05; Figure 4A). However, there were no significant differences in cholic acid (CA), lithocholic acid (LCA), chenodeoxycholic acid (CDCA) between the two groups (*p* > 0.05; Figure 4A). Co-occurring network analysis revealed a strong correlation between K01442 and the genera *Blautia*, *Lachnospira*, *Clostridium*, and *Peptostreptococcus* within the phylum Bacillota, as well as the genus *Eggerthella* in the phylum Actinomycetota, further supporting our findings (Figure 4B). Furthermore, cluster analysis suggests that an increase in the abundance of a specific microbial community may correlate with a rise in the number of microbial communities exhibiting similar characteristics or behaviours (Figure 4C).

### 3.6. Impact of Swollenin on Nitrogen Cycling in the Intestine of Young Goats

**Figure 5:**
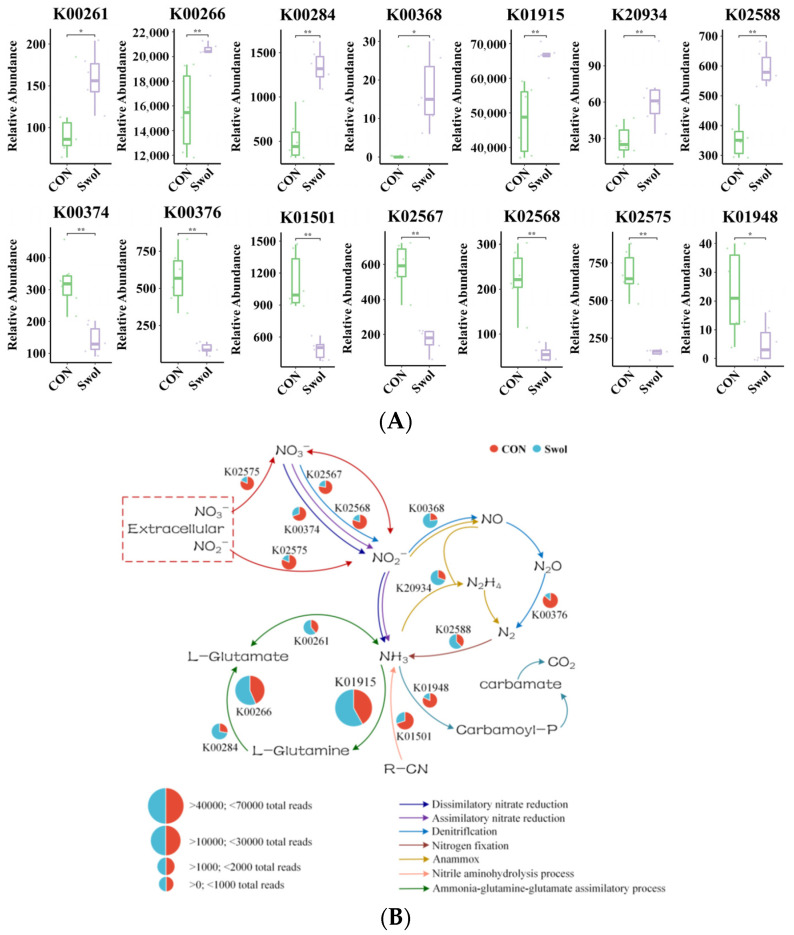
Effect of Swollenin on the significantly different abundance of genes related to nitrogen metabolism (**A**), and the proportion of gene readings for core functional orthologs and their main roles in the nitrogen metabolism pathway (**B**). Asterisk symbols indicate statistical significance: * (*p* < 0.05), ** (*p* < 0.01). Dots represent individual sample data points of each group. Lines with arrows in different colors represent distinct nitrogen metabolism processes. The size of the circles indicates the total reads of different ho-mologous gene clusters. The proportion of different colors in the circles represents the comparison of the two groups in terms of the total reads of each homologous gene cluster. CON, control group; Swol, swollenin group; NO_2_^−^, nitrite; NO_3_^−^, nitrate; NO, nitric oxide; N_2_O, nitrous oxide; N_2_, nitrogen; N_2_H_4_, hydrazine; NH_3_, ammonia; R-CN, nitrile; CO_2_, carbon dioxide.

The Wilcoxon test was employed to assess the abundance of genes associated with nitrogen metabolism (ko00910) across two groups, with significant gene enrichment displayed in Figure 5A. The findings from this experiment indicate that the addition of Swollenin substantially influences the nitrogen metabolism pathway within the intestines of young goats. Furthermore, the experiment visually represents the total reads of KOs in both groups through a pie chart, highlighting their primary functions in the nitrogen metabolism pathway (Figure 5B). Compared with the CON group, the enrichment level of *K02575* (nitrate/nitrite transporter, NRT2) in the microbial community of young goats was significantly lower following Swollenin supplementation. Meanwhile, the abundances of key genes involved in nitrogen metabolism pathways such as dissimilatory nitrate reduction and denitrification, including *K00374* (nitrate reductase γ subunit, narI), *K02567* (nitrate reductase, napA), and *K02568* (nitrate reductase electron transfer subunit, napB), were significantly lower in the Swollenin group compared with those in the CON group.

Interestingly, in the Swollenin group, the abundance of *K00368* (nitrite reductase, nirK) was significantly higher (*p* < 0.05), which may promote the conversion of nitrite (NO_2_^−^) to nitric oxide (NO). Meanwhile, the abundances of *K20934* (hydrazine synthase subunit) and *K02588* (nitrogenase iron protein, nifH) were significantly higher compared with those in the CON group (*p* < 0.05). In the nitrogen assimilation pathway, following Swollenin supplementation, the abundances of *K00261* (glutamate dehydrogenase, gdhA), *K01915* (glutamine synthetase, glnA), *K00266* (glutamate synthase small chain, gltD), and *K00284* (glutamate synthase, gltS) were all significantly higher.

### 3.7. The Co-Occurrence Network Among Nitrogen Metabolism Pathways, Secondary Bile Acid Metabolism-Related Genes, and Microbial Communities

**Figure 6:**
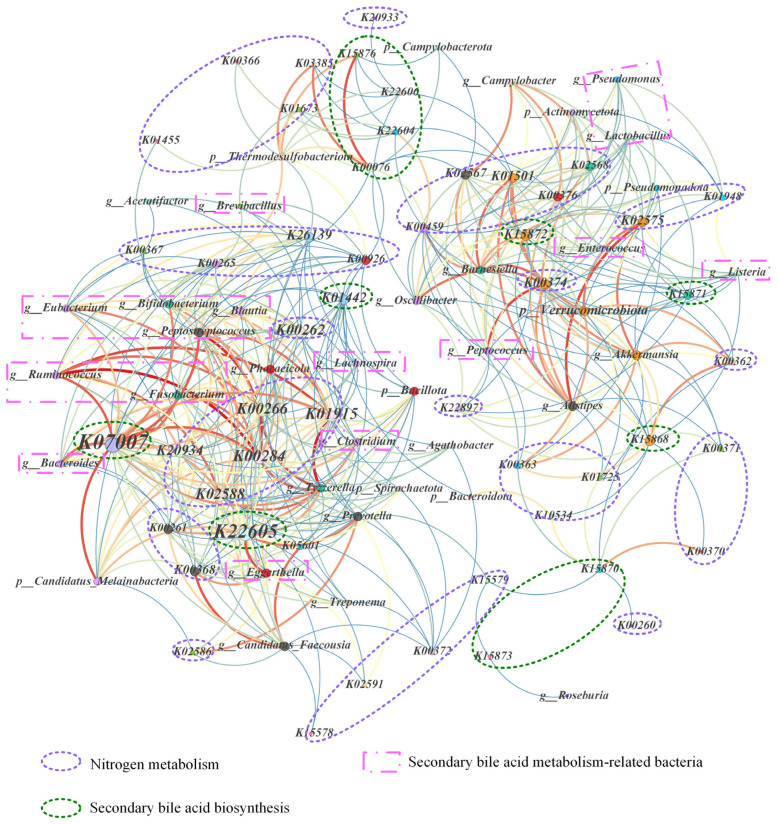
Co-occurrence network among the nitrogen metabolism pathway, secondary bile acid metabolism-related genes, and microbial communities. Nodes represent bacterial communities and core functional orthologs of nitrogen metabolism and secondary bile acid biosynthesis pathways. Node size and edges represent the degree of nodes and significant positive correlations (r > 0.5 and *p* < 0.05), respectively. The community detection algorithm (resolution of 1.0) resolves the overall data and assigns different colors to nodes and edges. The parts enclosed by dashed boxes in different colors represent the gene homologous clusters in nitrogen metabolism and secondary bile acid metabolism, as well as the secondary bile acid metabolism-related bacteria.

This study revealed a significant positive correlation between genes associated with secondary bile acid metabolism, such as *K07007*, *K22605*, and *K01442*, and key genera of gut microbiota, as well as important gene clusters involved in various nitrogen metabolism pathways (Figure 6). In this network visualization, 29 microbial communities from nine phyla have been identified as potential bacteria, which include 37 nitrogen metabolism-related gene clusters and 12 bile acid metabolism-related gene clusters. The Bacillota, Verrucomicrobiota and Candidatus Melainabacteria phyla are the primary groups harbouring multiple gene clusters pertinent to bile acid and nitrogen metabolism. Among the dominant phyla in both groups, the phylum Bacillota encompasses numerous genera that play crucial roles in bile acid metabolism. For instance, *Ruminococcus* is a potential host of *K07007* and *K22605*. Meanwhile, this study revealed that these genera also contribute to intestinal nitrogen metabolism—*Ruminococcus*, for example, is a potential host of nitrogen metabolism-related genes such as *K00261*, *K00266*, *K00284*, and *K01915*.

## 4. Discussion

Previous research has shown that Swollenin effectively disrupts fibrous structures, leading to a modest increase in reducing sugar release from xylan, Avicel, rice straw, wheat straw, and corn straw [14]. Notably, when co-administered with cellulolytic enzymes, Swollenin demonstrates significant synergistic effects [14]. These findings highlight the potential of Swollenin to enhance the enzymatic saccharification efficiency of agricultural crop residues. This enhancement provides innovative strategies to improve the utilization of lignocellulosic biomass by ruminants. The current trial data suggest that daily supplementation of 32 mg/d Swollenin did not adversely affect the growth performance of young goats. This aligns with findings from previous studies in lambs, and in goats and beef cattle, where fibrolytic enzyme additives generally improved feed conversion efficiency in young ruminants, although their effects on average daily gain often lacked statistical significance [19,20]. This suggests that the benefits of carbohydrate hydrolase for young ruminants mainly stem from optimized energy utilization, improved visceral organ development, and the establishment of long-term productivity potential, rather than merely stimulating appetite or achieving maximum short-term growth rates. However, future studies will require a larger number of experimental animals to further verify this hypothesis and explore the underlying deeper physiological regulatory processes.

Despite the minimal direct nutritional contribution of Swollenin at the current supplementation rate, the observed decrease in feed intake, combined with improved conversion efficiency, indicates enhanced digestive efficiency in young goats. These reallocated nutrients may provide a strong foundation for long-term gastrointestinal health and stability of the microbial ecosystem. Therefore, we hypothesize that Swollenin can influence gut microbiota structure and fermentation dynamics. The following section will explain how these microbial adaptations contribute to the modest yet sustained improvements in feed utilization observed in this study.

Figure 1A analysis highlighted compositional shifts among the top 10 most prevalent bacterial phyla in the gut microbiota of young goats following Swollenin consumption. Among them, the abundances of phyla Bacillota and Bacteroidota in the Swollenin group were higher than those in the CON group. Numerous microorganisms within the phylum Bacillota secrete extracellular hydrolytic enzymes like cellulases, lipases, and proteases, aiding hosts in nutrient utilization and energy acquisition [21,22]. The phylum Bacteroidota facilitates the breakdown of indigestible polysaccharides and a small fraction of cellulose, promoting organic matter fermentation [23]. Additionally, Bacillota bacteria play a pivotal role in intestinal bile acid conversion, particularly in catalyzing the production of biologically active secondary BAs. Furthermore, the phylum Bacteroidota is instrumental in bile acid dissociation and significantly impacts intestinal fecal nitrogen cycling, particularly ammonia production and urea utilization [24,25].

There is a greater abundance of phylum Spirochaetota in the Swollenin group. Microorganisms belonging to Spirochaetota can convert carbohydrates into simple volatile fatty acids for host utilization [26]. However, the abundances of Pseudomonas (formerly Proteobacteria) and Verrucomicrobiota in the Swollenin group were lower. The phylum Pseudomonas, also recognized as the phylum Proteobacteria, includes symbiotic bacterial species that contribute to healthy mammalian intestines [27]. However, these species do not constitute a significant portion of the natural gut microbiota [28]. Current literature widely supports the idea that a significant increase or gradual accumulation of the phylum Proteobacteria in the gut indicates instability or ecological imbalance within the gut microbiota community. In certain intestinal environments, these bacteria can transition into colonic bacteria that provoke inflammatory responses [29]. Certain microorganisms in the phylum Verrucomicrobiota are beneficial for maintaining glucose homeostasis, enhancing metabolism, and increasing intestinal mucus layer thickness [30,31]. Therefore, a deeper exploration of the gut microbiota changes in young goats following Swollenin intake is warranted.

The results reveal that after consuming Swollenin, the relative abundances of the genera *Bacteroides*, *Treponema*, *Ruminococcus*, *Clostridium*, *Prevotella*, and *Eubacterium* in the gut microbiota of young goats were higher. The *Bacteroides* and *Prevotella* genera are notable members of the phylum Bacteroidota, playing a crucial role in intestinal fecal nitrogen cycling. *Bacteroides ruminicola* has been shown to exhibit urease activity; by utilizing urea, it provides assimilable nitrogen for itself and other microbial members while also participating in the host’s nitrogen salvage process [32]. This dynamic exemplifies nitrogen assimilation pathways in the animal gut and enhances our understanding of the complex regulatory networks governing nitrogen metabolism within the phylum Bacteroidota [25]. The genera *Ruminococcus*, *Clostridium*, and *Eubacterium*, prominent in the phylum Bacillota, contribute significantly to gut health. For instance, the *Clostridium* catalyses the conversion of primary BAs to secondary BAs through an enzyme system encoded by its unique bile acid-induced (*bai*) gene cluster, including 7α-dehydroxylase [24]. However, the abundance of the genus *Akkermansia* was lower in the Swollenin group. This genus is a dominant genus within the phylum Verrucomicrobiota of the goat gut microbiota and has garnered attention for its roles in mucosal health, energy metabolism, and inflammatory markers [33].

The observed changes in gut microbiota abundance at the genus level following Swollenin consumption correspond with shifts in microbial community structure at the phylum level, as shown in Figure 1A. These alterations in microbial communities may result from competition among symbiotic microorganisms in the host gut for nutrients, such as nitrogen sources, driven by metabolic activities that provide a collective competitive advantage [34]. Consequently, the ingestion of Swollenin by young goats influences microbial communities occupying various ecological niches, leading to alterations in community structure. This competitive dynamic may significantly affect the host’s physiological state, particularly influencing secondary metabolites in the gut [35].

The gut microbiota serves as a core mediator of host metabolism, with critical implications for the synthesis and biotransformation of BAs. Different microorganisms may significantly contribute to environmental changes and can serve as biomarkers for both the control and Swollenin groups. Genera such as *Bacteroides*, *Bifidobacterium*, *Blautia*, *Clostridium*, *Enterococcus*, *Lactobacillus*, and *Listeria* produce BSH, a key enzyme in secondary bile acid synthesis [16]. BSH catalyzes the conjugation of primary BAs, playing a pivotal role in the microbial modification of BAs by converting taurine and glycine in bound BAs into free BAs [36]. Given that both *Bacteroides* and *Clostridium* are dominant bacteria in the gut of young goats (with abundances greater than 1%, as shown in Figure 1D), and that *Bifidobacterium* is a vital core component of the physiological microbiota in animal intestines, it is hypothesized that after consuming Swollenin, the dissociation of primary BAs in the intestine will increase. This could lead to a rise in the content of free BAs, potentially promoting intestinal health.

Concomitantly, the genera *Bacteroides*, *Ruminococcus*, *Eggerthella*, *Peptostreptococcus*, *Clostridium*, *Eubacterium*, *Blautia*, and *Lachnospira*, which contain *bai* genes, can produce the hydroxysteroid dehydrogenase (HSDH) family [37,38,39,40]. HSDHs facilitate the reversible oxidation of the 3-, 7-, and 12-hydroxyl groups of conjugated BAs, ultimately resulting in isomerization [16,41]. Both α-HSDHs and β-HSDHs contribute to this isomerization process, thereby mitigating the toxicity of BAs and safeguarding the host’s normal metabolic functions [39]. *Clostridium* and *Ruminococcus* are capable of producing both 7α-HSDH and 7β-HSDH enzymes, which facilitate the conversion of CDCA to UDCA through isopropylation [39,42]. However, UDCA can also be synthesized from CA via a two-step process involving isopropylation and 12α-HSDH oxidation [39]. This may be the reason why the gene abundance of the secondary bile acid biosynthesis pathway in the gut of young goats was significantly higher after Swollenin intake.

The abundance of genes *K07007* and *K22605* is significantly higher in the Swollenin group, while the abundance of gene *K01442* is also higher but without a significant difference. From the secondary bile acid biosynthesis pathway (ko00121, Figure 3), we infer that *K07007* functions as a 3-dehydro-bile acid δ4,6-reductase in the bile acid 7α-dehydroxylation pathway, catalyzing two consecutive reductions of double bonds in the A/B rings of bile acids following 7α-dehydration [38]. This process can potentially increase the production of Lithocholyl-CoA in the intestinal environment and promote the synthesis of lithocholic acid (LCA). *K22605* can modify secondary bile acids into 3β-bile acids (also known as iso-bile acids) via a 3-oxo intermediate [43]. *K01442*, also known as BSH, plays a crucial role in the biotransformation of primary BAs in the intestine [44]. This transformation is predominantly executed by intestinal bacteria containing BSHs, which convert conjugated BAs into their unconjugated forms [45]. Notably, the abundances of the genera *Bacteroides* and *Bifidobacterium* in the gut of young goats in the Swollenin group were significantly higher. This may be one of the reasons for the increased abundance of *K01442*, which is consistent with the previously stated view that a variety of intestinal microorganisms have the ability to produce BSH.

The abundance of genes *K15868*, *K15871*, and *K15872* is significantly higher in the CON group. *K15868* and *K15871* are genes that encode key enzymes involved in converting BAs into their acyl-CoA thioesters [37]. *K15872* functions as a bile acid 7α-dehydratase that participates in the 7-dehydroxylation process associated with bile acid degradation [46]. This study demonstrates that Swollenin holds promise for increasing the abundance of functional genes associated with the secondary bile acid biosynthesis pathway in mammals, thereby promoting the diversification of secondary bile acids in the animal gut.

The BAs serve not only as a communication bridge between the host and gut microbiota but also play a vital role in multiple physiological processes, including biological metabolism. This study found that Swollenin intake by young goats promotes the metabolism of four primary BAs while simultaneously elevating UDCA and DCA levels, two secondary BAs. The increased dissociation of primary BAs correlates with enhanced resistance to bile toxicity [16,47]. Notably, despite the significant increase in various bile acid-metabolizing microorganisms in the goat gut, such as *Bacteroides*, *Ruminococcus*, and *Bifidobacterium*, and a corresponding rise in the abundance of the homologous gene cluster *K01442* encoding BSH, the differences between the Swollenin group and the CON group were not significant. This phenomenon may be attributed to the presence of BSH-encoding genes across multiple gut microbiota [16,48]. Previous research has demonstrated that BSH can inhibit the expression of virulence genes in *Vibrio cholerae* by hydrolyzing TCA, which in turn reduces the colonization of diarrheal pathogens in the intestine [49]. In young goats, the intake of Swollenin significantly lowered the levels of TCA, GCA, TCDCA, and GCDCA in their intestines. This reduction is crucial for maintaining bile acid balance and promoting overall intestinal health.

Additionally, in the Swollenin group, the levels of UDCA and DCA in the goat intestine significantly increased (Figure 4A). UDCA enhances bile stasis by promoting hepatic BAs excretion and reducing intestinal BAs reabsorption [50]. DCA may stimulate the growth of beneficial gut bacteria, thereby providing protection against specific pathogens, such as inhibiting *Clostridium difficile* growth [51,52]. This antimicrobial property underscores the importance of DCA in maintaining gut health and preventing infections. Evidence also suggests that DCA can inhibit the growth of gallbladder cancer growth by modulating microRNA maturation and influencing key signalling pathways involved in cell proliferation [53]. Thus, maintaining an appropriate level of DCA in the gut could be an effective strategy for enhancing disease prevention and control mechanisms.

In summary, after young goats consumed Swollenin, the microbial community richness was higher, and the number of functional homologous genes associated with secondary bile acid biosynthesis in the gut increased significantly. This may contribute to the increased dissociation of primary BAs, influenced by the synergistic effects of diverse microorganisms, particularly those in the phylum Bacillota (Figure 4B). Figure 4C shows an increase in the abundance of specific microbial communities with synergistic effects. This led to enhanced gene enrichment and the production of differentiated metabolic products within the community, reflecting the active participation of microbial communities in metabolic cross-feeding [54]. These findings deepen our understanding of how functional orthologs are regulated within microbial populations and contribute to the broader field of bile acid metabolism regulation by exploring the relationships between various Bacillota bacteria and specific genes, offering potential targets for future therapeutic interventions and diagnostic methods for bile acid metabolism diseases. However, future studies investigating the regulation of the abundance of bile acid metabolism-related genes (e.g., *K01442*) through nutritional interventions that modulate the microbial structure in the animal gut may be further validated by increasing the sample size, establishing in vitro metabolic models, and conducting gut microbiota perturbation studies.

Previous studies have indicated that increased secondary bile acid content in the intestine boosts protease activity in aquatic animals by 30–50% [55]. BAs have also been shown to optimize the microenvironment for fat digestion through emulsification, which indirectly enhances protein hydrolysis efficiency [56,57]. Overall, alterations in bile acid metabolism patterns may interfere with essential cellular processes, including the activation status of nitrogen metabolism-related pathways. Currently, no research has established a link between changes in gene enrichment related to nitrogen metabolism in ruminants and increased bile acid metabolism. Therefore, this study will further investigate the enrichment status of intestinal fecal nitrogen metabolism-related genes in young goats after Swollenin intake.

The regulation of intestinal nitrogen metabolism in young goats by Swollenin is reflected in altered enrichment of nitrogen cycle-related genes, indicating that it exerts multifaceted effects on nitrogen utilization and cycling. The abundances of *K02575*, *K00374*, *K02567*, and *K02568* were significantly lower in the Swollenin group. *K02575* facilitates the uptake of nitrate (NO_3_^−^) and NO_2_^−^ from the environment into cells [58]. *K02567* and *K02568* form the nitrate reductase NapAB complex, both capable of converting NO_3_^−^ and NO_2_^−^*,* respectively [59,60]. Nitrogen is a crucial nutrient for all life forms on Earth, yet its availability varies widely. The number of microorganisms that can directly fix nitrogen is minimal; most rely on reactive nitrogen compounds like ammonium salts and nitrates, for growth. Dissimilar nitrate reduction is the first step in the denitrification process. Additionally, the increased denitrification activity of microorganisms accelerates the production of nitric oxide (NO), nitrous oxide (N_2_O), and nitrogen (N_2_) in the fermentation environment [61]. It is hypothesized in this study that the ingestion of Swollenin can reduce the uptake of NO_3_^−^ and NO_2_^−^ by the gut microbiota of goats, and this change in uptake capacity may further inhibit the initiation of denitrification processes. It has been established that NO_3_^−^ and NH_4_^+^ serve as primary inorganic nitrogen sources for plants; however, using NH_4_^+^ as the sole or predominant nitrogen source can result in ammonium toxicity [62]. Interestingly, NO_3_^−^ found in nitrate nitrogen fertilizers has been shown to effectively mitigate ammonium toxicity, thereby enhancing nitrogen use efficiency in crops [62]. Ensuring the normal physiological functions of animals, while minimizing denitrification and preventing excessive conversion of NO_3_^−^ and NO_2_^−^ in the intestine can facilitate the production of high-quality nitrogen fertilizers from animal feces.

For microorganisms that utilize NO_2_^−^, the ingestion of Swollenin resulted in a significant increase in the abundance of key genes associated with nitrogen fixation and assimilation, such as *K00368*, *K20934* and *K02588*. This process enhances the conversion of NO_2_^−^ to ammonia (NH_3_), representing a critical step in the nitrogen fixation pathway [4]. In the nitrogen assimilation pathway, the enrichment of *K00261*, *K01915*, *K00266*, and *K00284* likely promotes the synthesis of L-glutamate. In summary, the enrichment levels of these genes were significantly higher, which may collectively improve the nitrogen fixation and assimilation efficiency in the gut of young goats.

Microorganisms exhibit sensitive responses to environmental changes. For instance, gut microbiota can adjust their behaviour by sensing their surrounding environment through quorum sensing mechanisms [63]. This study demonstrates that the physiological activity of Swollenin can regulate the microbial composition in the gut of young goats, thereby influencing the gene enrichment of nitrogen metabolism-related enzymes. These targeted effects provide new insights for regulating animal nitrogen cycling and improving nitrogen utilization in the livestock industry. This knowledge can guide the design of more effective agricultural practices and contribute to sustainable nitrogen management.

The levels of secondary BAs not only regulate microbial activity but also impact the activity of various intestinal enzymes, potentially affecting nitrogen utilization by altering the composition and functionality of microbial communities responsible for nitrogen assimilation or ammonification [11,17]. Next, the symbiotic relationships between bile acid metabolism-related genes and nitrogen-cycling pathways within microbial communities will be explored. This will enhance our understanding of microbial community dynamics and nutrient cycling.

Co-occurrence network analysis reveals that the genus *Ruminococcus*, a core fiber-degrading bacterium in the gastrointestinal tract of ruminants, hosts the *K07007* and *K22605* gene clusters involved in bile acid metabolism. Moreover, this study identified the *Ruminococcus* as a potential host for 10 gene clusters related to nitrogen metabolism, including *K00261*, *K00266*, *K00284*, and *K01915*. This indicates a substantial increase in the abundance of the *Ruminococcus*, which not only affects bile acid metabolism in the animal intestine but also participates in crucial nitrogen-cycling processes, such as nitrogen assimilation, nitrogen fixation, and assimilatory nitrate reduction. The significant increase in the *K07007* gene cluster within the gastrointestinal tract may consequently enhance nitrogen metabolism processes in microorganisms, thereby facilitating assimilatory nitrate reduction and denitrification. Notably, the genus *Clostridium*, another dominant bacterial genus within the phylum Bacillota, is recognized for promoting cellulose degradation and improving feed digestibility [64]. *Clostridium aminophilum*, a species within this genus, plays a role in protein deamination [65]. This experiment revealed that the *Clostridium* genus serves as a potential host for gene clusters *K00261*, *K00368*, *K00284*, *K00266*, *K20934*, *K01915*, *K00926*, *K05601*, and *K02588*, which are linked to nitrogen metabolism pathways, including denitrification, anammox, and nitrogen fixation. Meanwhile, the genus *Clostridium* hosts the *K07007*, *K22605*, and *K01442* gene clusters related to bile acid metabolism, facilitating the biotransformation of primary BAs in the intestine and playing a crucial role in enriching secondary BAs. The significant positive correlations among *K07007*, *K22605*, and *K01442* with gene clusters related to assimilatory nitrate reduction, nitrogen fixation, anammox, denitrification, and ammonia utilization suggest a more integrated understanding of the genus *Clostridium*’s role in both nitrogen and bile acid metabolism processes [64,65]. Therefore, this study expands on existing knowledge by explicitly linking these genes.

The genus *Bacteroides*, a prominent member of the phylum Bacteroidota, is recognized for its extensive genome and remarkable metabolic flexibility. It excels at breaking down various carbohydrates and plays a critical role in transforming proteins, amino acids, BAs, and other substances within the intestine [15]. Research indicates that *Bacteroides* is not only a significant producer of BSH but also contributes to BAs isomerization [66]. Our study reveals a strong positive correlation between the *Bacteroides* genus and the *K07007* and *K22605* gene clusters, consistent with previous findings. Furthermore, *Bacteroides* are involved in endogenous nitrogen recycling and demonstrate robust amino acid metabolism capabilities [32,67]. However, there is currently no direct experimental evidence linking the metabolic activities of *Bacteroides* to the regulation of nitrogen metabolism in ruminants via the bile acid signalling pathway. As illustrated in Figure 6, the genus *Bacteroides* may act as a potential reservoir for gene clusters associated with nitrogen fixation, assimilatory nitrate reduction, anammox, and denitrification pathways. Notably, it shows a significant positive correlation with several nitrogen fixation-related genes, including *K01915*, *K00284*, *K00266*, *K02588*, *K00262*, *K00265*, and *K00261*. This underscores the *Bacteroides* genus’s role in shaping gut metabolite profiles and influencing the nitrogen balance in hosts.

Similarly, the genus *Candidatus Faecousia*, a dominant bacterial group within the phylum Bacillota, is recognized as a key acetate-producing bacterium in the gastrointestinal tract of ruminants, playing a vital role in regulating systemic energy metabolism [15]. To date, there is no research confirming that the *Candidatus Faecousia* genus possesses the ability to convert BAs in the ruminant gut. Given its classification within the Oscillospiraceae family, which shares close phylogenetic and ecological ties with the Ruminococcaceae family, it is plausible to hypothesize that *Candidatus Faecousia* may possess similar genetic capabilities for bile acid metabolism from an evolutionary standpoint. As shown in Figure 6, *Candidatus Faecousia* is significantly positively correlated with gene clusters *K22605*, *K07007*, and *K01442*. Meanwhile, similar to bile acid metabolism, there is a conspicuous lack of studies addressing nitrogen metabolism phenotypes in the *Candidatus Faecousia* genus. Our findings demonstrate that this genus is positively associated with nitrogen metabolism gene clusters, including *K00368*, *K05601*, *K00266*, *K00261*, *K01915*, *K00284*, *K20934*, *K02586*, *K02588*, *K15578*, *K00372*, and *K02591*. It is speculated that, as members of the Oscillospiraceae family, the *Candidatus Faecousia* genus may participate in the metabolism of proteins or amino acids, thus providing nitrogen sources for other rumen microorganisms [68].

Previous research has highlighted the significance of animal bile acid metabolism in various cellular processes [16,56]. This study builds upon that foundation by explicitly linking specific genes to nitrogen metabolism through bacterial communities. It is hypothesized that mechanisms of bile acid metabolism may regulate the expression levels of nitrogen-cycling enzymes, thereby influencing nitrogen utilization in animals. This research contributes to the understanding of the interactions between intestinal metabolites and cellular functions. However, our study has certain limitations, including the undetermined optimal dosage of Swollenin in the production of young ruminants and its stability in the gastrointestinal tract, as well as the relatively small sample size used in metagenomic sequencing. Meanwhile, further validation through larger-scale and more diverse studies is required. For instance, co-culturing Swollenin with bile acid-metabolizing bacterial strains and detecting the activity of key bile acid-metabolizing enzymes will provide comprehensive evidence for Swollenin-induced intestinal metabolic changes. Therefore, future research should explore the potential mechanisms underlying the observed correlations and further evaluate the practical significance and application effects of these results.

## 5. Conclusions

In conclusion, the introduction of Swollenin has increased the richness of gut microbiota in young goats, optimized microbial structure, and improved feed conversion efficiency. This enhancement not only influences the generation of secondary metabolites in the animal intestine but also significantly impacts the host’s physiological state. The increased abundance of bile acid metabolism-related bacteria significantly enhanced the gene enrichment level of the secondary bile acid biosynthesis pathway in the intestines of young goats. Additionally, the dissociation of primary BAs has improved, fostering greater diversity of secondary bile acids within these animals’ intestines. Moreover, Swollenin addition regulated nitrogen metabolism pathways in young goats, especially reflected by significantly higher abundances of nitrogen fixation and assimilation genes. Our findings suggest a potentially strong correlation between bile acid metabolism and nitrogen metabolism pathways. This highlights the complex symbiotic, co-metabolic, and co-evolutionary relationships between hosts and their gut microbiota, as well as the adaptive mechanisms among various organisms. This research enhances our understanding of geochemical element cycling driven by gut microbiota. Targeted intervention measures should be formulated to optimize the symbiotic relationship between the host and the gut microbiota in response to the sustainable development strategy of the animal husbandry industry.

## Figures and Tables

**Table 1:** Effects of dietary Swollenin on growth performance of goats (mean ± SD).

Item ^1^	Control	Swollenin	*p*-Value
Initial BW (kg)	6.06 ± 0.61	6.26 ± 0.35	0.364
Final BW (kg)	8.12 ± 0.54	8.43 ± 0.25	0.104
ADG ^2^ (g d^−1^)	68.57 ± 6.69	72.47 ± 8.14	0.290
ADFI ^3^ (g d^−1^)	273.63 ± 5.77	263.08 ± 11.40	0.049
Feed:gain (F/G)	4.02 ± 0.31	3.67 ± 0.40	0.082

^1^ BW, body weight; ADG, average daily gain; ADFI, average daily feed intake. ^2^ ADG was calculated over a 30-day feeding period. ^3^ Dry-matter basis.

## Data Availability

The raw data supporting the conclusions of this article will be made available by the authors on request.

## Supplementary Materials

The following supporting information can be downloaded at: https://www.mdpi.com/article/10.3390/ani16010149/s1, Table S1: Basal Diet Formulation for Young Goats (DM).

## Abbreviations

The following abbreviations are used in this manuscript:

BAs	Bile acids	ADFI	Average daily feed intake
BSH	Bile salt hydrolase	Swol	Swollenin group
CON	Control	BW	Body weight
ADG	Average daily gain	GCA	Glycocholic acid
DMI	Daily dry matter intake	TCA	Tarocholic acid
F/G	Feed conversion ratio	GCDCA	Glycochenodeoxycholic acid
KEGG	Kyoto Encyclopedia of Genes and Genomes	TCDCA	Taurochenodeoxycholic acid
KO	KEGG Orthology	CA	Cholic acid
FDR	False discovery rate	CDCA	Chenodeoxycholic acid
LDA	Linear Discriminant Analysis	DCA	Deoxycholic acid
LEfSe	Linear Discriminant Analysis Effect Size	LCA	Lithocholic acid
PCoA	Principal Coordinate Analysis	UDCA	Ursodeoxycholic acid
NO_2_^−^	Nitrite	NO_3_^−^	Nitrate
NO	Nitric oxide	N_2_O	Nitrous oxide
N_2_	Nitrogen	N_2_H_4_	Hydrazine
NH_3_	Ammonia	R-CN	Nitrile
CO_2_	Carbon dioxide	*bai*	Bile acid-induced
HSDH	Hydroxysteroid dehydrogenase		
