# Supplementary material for: Synergistic Regulation of Bile Acid-Driven Nitrogen Metabolism by Swollenin in Ruminants: A Microbiota-Targeted Strategy to Improve Nitrogen Use Efficiency
Source: Animals (Basel). 2026 Jan 5;16(1):149. doi: 10.3390/ani16010149 (PMC12784710; doi:10.3390/ani16010149)
Supplement: Supplementary file 1 [file animals-16-00149-s001.zip › animals-4019682-supplementary.pdf]

Table S1. Basal Diet Formulation for Young Goats (DM).

Ingredient composition	Content (%)	Nutrient Levels (DM basis) [2]	Content (%)
Alfalfa hay	28.00	Crude Protein (CP)	19.60
Soybean meal	24.50	Crude Fat (EE)	5.68
Corn	22.90	Dry Matter (DM)	95.25
Whey powder	16.00	Neutral Detergent Fiber (NDF)	25.90
Fatty powder	5.00	Acid Detergent Fiber (ADF)	17.90
Calcium carbonate ($CaCO_3$)	0.50	Calcium (Ca)	1.06
Dibasic calcium phosphate ($CaHPO_4$)	1.50	Phosphorus (P)	0.68
NaCl	0.60		
Premix[1]	1.00		
Total	100		

Note: [1]Premix provides the following per kg of concentrate: $FeSO_4 \bullet 7H_2O$ 2.5 g, $CuSO_4 \bullet 5H_2O$ 0.8 g, $MnSO_4 \bullet H_2O$ 3 g, Na_2SeO_3 10 mg, KI 40 mg, $CoCl_2 \bullet 6H_2O$ 30 mg, vitamin A 95,000 IU, vitamin D 17,500 IU, vitamin E 18,000 IU. [2]All nutrient levels were the measured value.
